# Low-PSMA hormone-sensitive prostate cancer: a distinct clinical and molecular state

**DOI:** 10.1186/s10020-026-01546-w

**Published:** 2026-07-03

**Authors:** Haotian Wu, Ang Li, Zhou Jiang, Weiwei Zhang, Kai Shen, Bo Liu, Ruopeng Su, Xinyu Liu, Xinyu Chai, Tianxiang Wang, Junyi Wang, Jiayan Yi, Xiang Zhou, Yinjie Zhu, Dengfeng Cao, Qi Wang, Jiahua Pan, Wei Xue

**Affiliations:** 1 Department of Urology, Ren Ji Hospital, Shanghai Jiao Tong University School of Medicine, Shanghai, China; 2 Department of Pathology, Ren Ji Hospital, Shanghai Jiao Tong University School of Medicine, Shanghai, China; 3 Department of Nuclear Medicine, Ren Ji Hospital, Shanghai Jiao Tong University School of Medicine, Shanghai, China; 4 Shanghai Key Laboratory for Tumor Microenvironment and Inflammation, Shanghai Jiao Tong University School of Medicine, Shanghai, China

**Keywords:** Hormone-sensitive Prostate Cancer, Prostate-specific Membrane Antigen, Proteomic Analysis, Tumor Biomarker

## Abstract

**Background:**

A subset of hormone-sensitive prostate cancer (HSPC) exhibits low prostate-specific membrane antigen (PSMA) uptake on PET imaging, which may limit the performance of PSMA-based detection and PSMA-directed therapeutic strategies. However, the clinical features and molecular basis of HSPC with low PSMA expression remain incompletely defined.

**Methods:**

We first analyzed a retrospective dual-tracer PET cohort to estimate the frequency and clinical characteristics of tumors with low PSMA uptake at initial diagnosis. We then collected radical prostatectomy specimens for proteomics profiling, classifying tumors as low-PSMA (*N* = 16) or high-PSMA (*N* = 19) based on concordant [^68^Ga]Ga-PSMA-11 PET and PSMA immunohistochemistry, with adjacent benign tissues included as controls. Label-free quantitative proteomics and integrative bioinformatic analyses were used to characterize low-PSMA–associated protein features and nominate candidate cell-surface membrane targets, followed by validation using RT-qPCR and immunohistochemistry.

**Results:**

Low-PSMA HSPC was identified in 7.23% (122/1,688) of patients in the retrospective dual-tracer PET cohort, indicating that reduced PSMA uptake is already observable at initial diagnosis. Although these tumors showed reduced PSMA expression, they were not uniformly low-risk, with 42.62% of cases exhibiting Gleason score ≥ 8. In the proteomic cohort, low-PSMA tumors showed concordant reductions in PSMA uptake and protein expression, supporting imaging–pathology alignment. Proteomic analysis revealed a distinct molecular state characterized by enrichment of tissue remodeling, repair, epithelial organization, and microenvironment-related programs, together with attenuation of androgen-response, E2F, and MYC-associated proliferative signaling. Cell-surface membrane surface target prioritization identified ALCAM and TACSTD2 as candidates, among which ALCAM was consistently upregulated in low-PSMA tumors at both mRNA and protein levels.

**Conclusions:**

HSPC with low PSMA expression represents a distinct clinical and molecular state rather than a simple low-risk subgroup. Its proteomic features suggest altered epithelial differentiation, tissue remodeling, and reduced canonical prostate cancer signaling. ALCAM may serve as a complementary membrane biomarker for improving the molecular recognition of low-PSMA HSPC and supporting future alternative imaging or targeting strategies.

**Supplementary Information:**

The online version contains supplementary material available at https://doi.org/10.1186/s10020-026-01546-w.

## Introduction

Prostate cancer is one of the most commonly diagnosed malignancies in men and a leading cause of cancer related mortality worldwide (Bray et al. 2024). Because treatment selection and prognosis are largely determined by metastatic burden and tumor aggressiveness, accurate staging at initial diagnosis is essential (Sweeney et al. 2015; Humphrey 2004; Parker et al. 2018). In this situation, prostate-specific membrane antigen (PSMA) has become a central molecular target for both imaging and therapy. PSMA-targeted PET/CT or PET/MR improves lesion detection compared with conventional imaging and has been rapidly integrated into clinical practice for primary staging (Sartor et al. 2021; Kiess et al. 2015). PSMA expression also guides patient selection for PSMA-directed radioligand therapy, underscoring its clinical importance as both a biomarker and a therapeutic target (Sartor et al. 2021).

However, PSMA expression is heterogeneous across prostate cancer. A subset of tumors shows low uptake on PSMA PET, creating a clinically important blind spot for PSMA based imaging and targeting. Lesions with reduced PSMA signal may be undetected, potentially resulting in incomplete disease assessment and suboptimal management (Ferraro et al. 2020; Derlin et al. 2023; Chen et al. 2025). Importantly, low PSMA uptake does not necessarily define a low-risk phenotype (Li et al. 2026). Emerging evidence suggests that reduced PSMA expression may accompany aggressive tumor states marked by lineage plasticity, dedifferentiation, or alternative oncogenic signaling. Yet most mechanistic insights and clinical discussion have centered on advanced prostate cancer, particularly castration resistant prostate cancer (CRPC) and neuroendocrine prostate cancer (NEPC) (Dardenne et al. 2016; 2019; Gan et al. 2023; Qin et al. 2012; Sayar et al. 2023).

Whether a biologically distinct low-PSMA subgroup already exists at the hormone sensitive, treatment naive stage remains insufficiently understood. This question is clinically relevant because hormone-sensitive prostate cancer (HSPC) represents a key window for curative intent treatment and risk adapted intensification. Even if PSMA heterogeneity is generally less pronounced than in CRPC or NEPC, a nonnegligible subset of newly diagnosed patients may still harbor low-PSMA tumors (Ferraro et al. 2020; Woo et al. 2025; Paschalis et al. 2019). In such cases, reliance on PSMA based staging alone may fail to capture clinically significant disease (Incesu et al. 2025). Complementary imaging such as [^18^F]FDG PET can provide an orthogonal view of tumor biology by detecting lesions associated with glycolytic or inflammatory activity (Chen et al. 2022, 2025; Shen et al. 2021; Ergül et al. 2024). The observation that some low PSMA uptake lesions remain metabolically active and can be pathologically confirmed therefore highlights the need for alternative and robust biomarkers, ideally cell surface proteins, to improve the detection and characterization of low-PSMA HSPC (Li et al. 2026; Chen et al. 2025; Ferraro et al. 2020).

In this study, we investigated newly diagnosed HSPC patients undergoing baseline [^68^Ga]Ga PSMA11 PET/CT and identified a distinct subgroup with low PSMA uptake. To ensure that downstream analyses faithfully represented this phenotype, we integrated imaging findings with PSMA immunohistochemistry (IHC) and restricted molecular profiling to PET-IHC concordant specimens. We then performed label free quantitative proteomic profiling of low-PSMA tumors, high-PSMA tumors, and adjacent benign tissues to define global proteomic alterations, heterogeneity, and pathway level differences. Finally, through integrative analyses across independent datasets, we prioritized membrane surface proteins as candidate complementary biomarkers. Together, these analyses characterize the proteomic basis of low-PSMA HSPC at diagnosis and nominate a candidate membrane biomarker for improved detection and biological stratification of this clinically underrecognized subgroup.

## Materials and methods

### Study design and patient cohorts

This study combined a retrospective dual-tracer PET imaging cohort and a proteomic cohort to define the prevalence, clinical features, and biological characteristics of low-PSMA treatment-naive hormone-sensitive prostate cancer (HSPC), and to identify complementary membrane biomarkers for this subtype.

For the retrospective clinical cohort, we reviewed consecutive patients who underwent baseline dual-tracer PET imaging with [^18^F]FDG and [^68^Ga]Ga-PSMA-11 at our institution between September 1, 2021 and April 30, 2025. Patients were eligible if they had treatment-naive prostate cancer with pathological confirmation and complete clinical and imaging data. Patients were excluded if they had received any prior anti-tumor treatment before imaging, lacked pathological confirmation, or had incomplete key clinical or imaging information. After screening, treatment-naive patients with confirmed pathological results were included for the final analysis.

For the proteomic cohort, we included patients who underwent radical prostatectomy and had adequate tumor tissue available for proteomic profiling. To minimize classification bias, low-PSMA and high-PSMA tumors included in the proteomic analyses were selected based on imaging-pathology concordance, integrating baseline PSMA PET findings with PSMA immunohistochemistry (IHC) results. Matched adjacent non-tumor tissues were collected when available.

All experimental procedures were approved by the Ethics Committee of Ren Ji Hospital, Shanghai Jiao Tong University School of Medicine (RA-2025–897). The requirement for written informed consent was waived for the retrospective clinical analysis where applicable. Written informed consent was obtained from patients enrolled in specimen collection and experimental validation procedures according to institutional regulations.

### PET imaging and image analysis

All included patients underwent baseline dual-tracer PET imaging with [^18^F]FDG and [^68^Ga]Ga-PSMA-11 before initiation of any anti-tumor treatment. For [^18^F]FDG PET/MR, patients fasted for at least 6 h, and imaging was performed 50–60 min after intravenous injection of [^18^F]FDG at 3.7 MBq/kg. For [^68^Ga]Ga-PSMA-11 PET/CT, routine whole-body imaging was performed 50–60 min after intravenous injection of [^68^Ga]Ga-PSMA-11 at 1.85 MBq/kg. To improve pelvic lesion assessment and reduce urinary bladder interference, forced diuresis was routinely performed in all included patients, with intravenous administration of 20 mg furosemide, oral hydration with at least 500 mL water, and frequent voiding. Additional delayed pelvic imaging was acquired approximately 1.5 h after routine imaging, covering two bed positions centered over the bladder region.

PET images were reviewed independently by experienced nuclear medicine physicians who were blinded to the proteomic data. Intraprostatic lesions and metastatic lesions, when present, were assessed visually and semi-quantitatively. PSMA uptake was evaluated using the miPSMA scoring system. Tumors with miPSMA scores of 0–1 were classified as low-PSMA, whereas tumors with miPSMA scores of 2–3 were classified as high-PSMA. In selected analyses, PET-derived semi-quantitative parameters, including SUV_max_, were also recorded.

### Tissue sampling and histopathological evaluation

Radical prostatectomy specimens were processed according to standard pathological procedures. Representative tumor regions adjacent non-tumor tissues were selected by an experienced genitourinary pathologist based on hematoxylin and eosin (H&E)-stained sections. Tissue sampling prioritized regions with adequate tumor cellularity and clear histological annotation for downstream proteomic and validation analyses.

### Protein extraction and label-free quantitative proteomics

Tissue samples from low-PSMA tumors, high-PSMA tumors, and adjacent tissues were subjected to label-free quantitative proteomic profiling. Briefly, tissue specimens were lysed in protein extraction buffer containing protease inhibitors, homogenized, and centrifuged to remove debris. Protein concentrations were determined using a standard protein quantification assay. Equal amounts of protein from each sample were reduced, alkylated, and digested with trypsin according to standard protocols. Peptides were then desalted and analyzed by liquid chromatography-tandem mass spectrometry (LC–MS/MS) using a high-resolution mass spectrometry platform.

Raw mass spectrometry data were processed using DIA-NN software and searched against the human reference proteome database. Protein identification and quantification were performed under standard false discovery rate (FDR) control criteria. Normalized protein abundance matrices were generated for downstream analyses. Proteins with excessive missing values were excluded according to prespecified filtering criteria, and retained protein intensities were log2-transformed before statistical analysis.

### Proteomic data preprocessing and differential expression analysis

Proteomic data preprocessing and statistical analyses were performed in R (version 4.5.1). Principal component analysis (PCA) was performed on normalized protein abundance data to assess overall sample distribution and group separation.

Differential expression analysis was conducted to compare low-PSMA and high-PSMA tumors. Additional comparisons between tumor and adjacent tissues were also performed where relevant. Fold changes and P values were calculated for each quantified protein. Proteins with a *P* value < 0.05 and a fold change ≥ 1 or ≤ −1 were considered differentially expressed proteins (DEPs).

### Gene set enrichment and pathway-level analyses

To characterize the biological programs associated with the low-PSMA phenotype, gene set enrichment analysis (GSEA) was performed using the full ranked proteomic list rather than only DEPs. Proteins were ranked according to the direction and magnitude of differential expression between low-PSMA and high-PSMA tumors. Hallmark gene sets, as well as gene ontology (GO) and kyoto encyclopedia of genes and genomes (KEGG) pathway databases, were used as reference collections.

To assess sample-level pathway activity, single-sample GSEA was performed for selected hallmark pathways. Based on the enrichment results, pathways enriched in low-PSMA tumors were summarized into a remodeling/repair-related axis, whereas pathways downregulated in low-PSMA tumors were summarized into an androgen response/proliferation-related axis. Composite protein-program scores were calculated by averaging standardized expression values of proteins included in each program. A delta score was further defined as the remodeling/repair-like score minus the androgen response/proliferation-like score.

Leading-edge proteins from significantly enriched hallmark pathways were extracted and intersected with DEPs to identify core proteins contributing to the observed pathway enrichment patterns.

### Assessment of tissue composition and tumor purity

To evaluate whether the observed proteomic differences were confounded by tissue composition, stromal content, or immune infiltration, stromal score, immune score, ESTIMATE score, and inferred tumor purity were assessed for each sample using established computational approaches. These metrics were compared between low-PSMA and high-PSMA tumors. Spearman correlation analyses were performed to evaluate associations between inferred tumor purity and selected pathway scores.

### Candidate membrane protein prioritization

To identify candidate cell-surface biomarkers that may complement PSMA in low-PSMA HSPC, we prioritized membrane-associated proteins consistently enriched in low-PSMA tumors across multiple datasets. In our discovery proteomic dataset (PXD076661), proteins upregulated in low-PSMA tumors relative to adjacent benign tissues were first identified. Two external public proteomic datasets (PXD049446 and OEP004998) were then analyzed independently. In each dataset, tumor samples were ranked by PSMA expression, and the lowest quartile was defined as the low-PSMA group. Differential expression analysis was performed between low-PSMA tumors and adjacent tissues within each dataset. Proteins identified from these external comparisons were subsequently intersected with our in-house upregulated protein set and with annotated membrane protein databases. Candidates were prioritized according to membrane localization, reproducible dysregulation across datasets, and potential suitability for translational applications, including molecular imaging and targeted therapy.

### Public dataset analysis

Publicly available transcriptomic datasets of prostate cancer were used for external validation. In particular, the cancer genome atlas prostate adenocarcinoma (TCGA-PRAD) dataset was analyzed to compare the expression of candidate genes between tumor and adjacent normal tissues. Where applicable, pan-cancer expression profiles were also examined to evaluate the relative abundance of prioritized candidate genes across tumor types.

Public datasets were downloaded and processed using standard bioinformatic workflows. Gene expression comparisons between groups were performed using appropriate statistical tests depending on the data distribution and analysis context.

### Immunohistochemistry

Formalin-fixed, paraffin-embedded prostate tissue specimens were sectioned at 4 μm thickness. After deparaffinization and rehydration, antigen retrieval was performed in EDTA buffer (pH 8.0) by heat treatment. Sections were then blocked with 10% donkey serum and incubated overnight at 4 °C with primary antibodies. Detailed information on the antibodies used in this study is provided in Supplementary Table 1. After washing, the sections were incubated with the corresponding secondary antibodies for 60 min at room temperature. Immunoreactivity was visualized using diaminobenzidine, followed by hematoxylin counterstaining. Representative images were acquired using a Nikon light microscope.

For semiquantitative analysis with QuPath (0.5.1), protein expression was assessed using the H-score method. Briefly, the H-score was calculated by multiplying the staining intensity score (0, no staining; 1, weak staining; 2, moderate staining; and 3, strong staining) by the percentage of positively stained cells (0–100), yielding a total score ranging from 0 to 300.

### Quantitative real-time PCR

Total RNA was extracted from tissue samples using TRIzol Reagent (15,596,026, Invitrogen) according to the manufacturer’s instructions. RNA concentration and purity were measured using a NanoDrop 2000c spectrophotometer (Thermo Fisher Scientific). Complementary DNA was synthesized using a reverse transcription kit (R223-1, Vazyme, China). Quantitative real-time PCR was performed using SYBR Green Master Mix (Q711-02, Vazyme, China) on a LightCycler 480 Real-Time PCR System (Roche). Relative mRNA expression levels were calculated using the 2^−ΔΔCq^ method. Primer sequences are listed in Supplementary Table 1.

### Statistical analysis

All statistical analyses were performed using R (version 4.5.1) and GraphPad Prism (version 9.5.0), unless otherwise specified. Continuous variables were summarized as median and interquartile range (IQR) or mean ± standard deviation, as appropriate. Categorical variables were summarized as counts and percentages.

Between-group comparisons for continuous variables were performed using the Wilcoxon rank-sum test or Student’s t-test, as appropriate. Comparisons of categorical variables were performed using the chi-square test or Fisher’s exact test. Univariable and multivariable logistic regression analyses were used to identify clinical factors associated with the low-PSMA phenotype. Odds ratios (ORs) and 95% confidence intervals (CIs) were reported. Continuous variables (normally distributed) were compared using two-tailed Student’s t-tests (two groups) or one-way ANOVA with Dunnett’s post hoc test (multiple groups); nonparametric data were analyzed via Mann–Whitney U or Kruskal–Wallis tests. Correlations were assessed using Spearman’s rank correlation coefficient. All statistical tests were two-sided, and *P* < 0.05 was considered statistically significant unless otherwise specified.

## Results

### Clinical characterization of low-PSMA hormone-sensitive prostate cancer (HSPC)

**Fig. 1:**
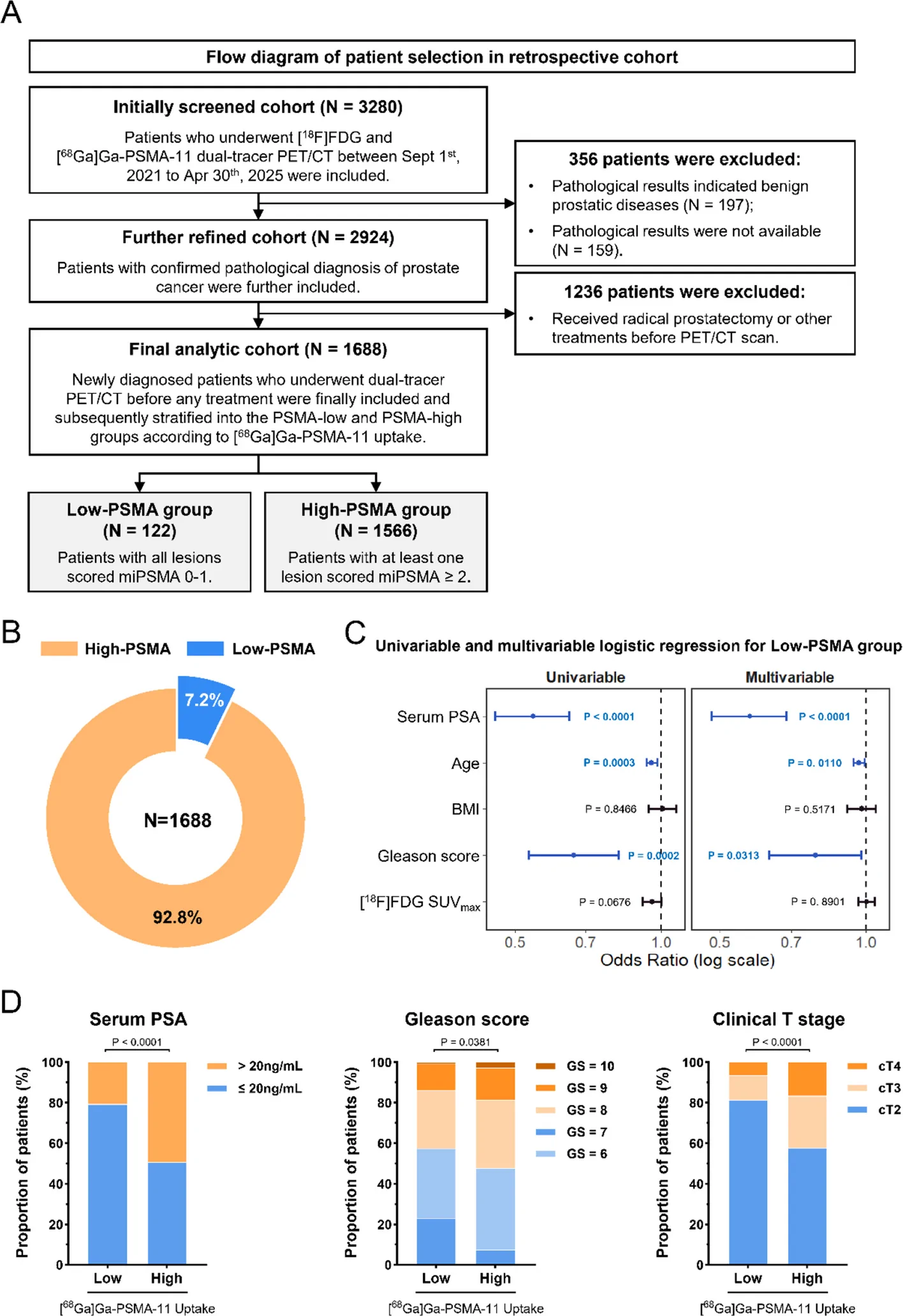
Cohort overview and clinical characteristics of low-PSMA prostate cancer at initial diagnosis. **A** Flow diagram of patient inclusion and exclusion for the retrospective dual-tracer PET cohort. **B** Distribution of low-PSMA and high-PSMA groups in the final cohort (*N* = 1,688), showing that low-PSMA group constitutes a distinct minority subset at initial diagnosis. **C** Univariable and multivariable logistic regression analyses identifying clinical variables associated with low-PSMA group. Odds ratios (ORs) and 95% confidence intervals (CIs) are shown for all variables included in the models. **D** Comparison of baseline clinicopathologic features between the low-PSMA and high-PSMA groups, including serum PSA level (≤ 20 vs > 20 ng/mL), Gleason score, and Clinical T stage. Low-PSMA tumors are associated with lower PSA levels, lower clinical stage, and a shift toward lower Gleason grade, while retaining a substantial proportion of high-grade disease. Statistical procedures are described in the Methods. *P* < 0.05 was considered statistically significant

Although PSMA heterogeneity is typically more pronounced in advanced disease states such as castration-resistant or neuroendocrine prostate cancer, a distinct subset of treatment-naive hormone-sensitive prostate cancer (HSPC) already exhibits low PSMA uptake at initial diagnosis. To define the prevalence and clinical features of this phenotype, we analyzed 1,688 HSPC patients from a dual-tracer PET cohort (Fig. 1A). Using miPSMA criteria, 122 patients (7.23%) were classified as low-PSMA, whereas the majority (92.77%) were high-PSMA (Fig. 1A–B; Table 1), establishing that low-PSMA disease is present early in prostate cancer evolution. We next compared baseline clinical characteristics between the two groups. Low-PSMA patients were younger and presented with significantly lower serum PSA levels at diagnosis (Table 1). They also showed a shift toward lower Gleason grades, with a higher proportion of Gleason score ≤ 7 and a reduced frequency of Gleason score ≥ 8 relative to high-PSMA patients. However, high-grade prostate cancer remained common, with 42.62% of low-PSMA patients exhibiting Gleason score ≥ 8, indicating that low PSMA uptake does not define a low-risk subtype. Consistent with this, low-PSMA patients had lower clinical T stage and a higher frequency of node-negative stage, whereas the incidence of metastatic stage was comparable between groups (Table 1; Fig. 1D). Multivariable logistic regression identified lower serum PSA, younger age, and lower Gleason score as independent features associated with the low-PSMA phenotype, while other clinicopathologic variables were not independently retained (Fig. 1C).

**Table 1:** Baseline clinical characteristics of the retrospective dual-tracer imaging cohort

Clinical Characteristics	Total	Low-PSMA	High-PSMA	*P* Value
	(*N* = 1688)	(*N* = 122)	(*N* = 1566)	
Age (y, Median, IQR)	70 (65.00–75.00)	68 (62.00–72.00)	70 (66.00–75.00)	0.0003
BMI (kg/m^2^, Median, IQR)	23.94 (21.97–25.76)	23.88 (22.15–25.61)	23.94 (21.97–25.77)	0.8456
Serum PSA (ng/ml, Median, IQR)	18.97 (10.02–46.02)	9.32 (5.86–17.37)	20.00 (10.68–44.45)	< 0.0001
Gleason Score (n, %)				0.0381
≤ 7	816(48.34%)	70(57.38%)	746(47.64%)	
≥ 8	872(51.66%)	52(42.62%)	820(52.36%)	
Clinical T Stage (n, %)				< 0.0001
T2a-c	1004(59.48%)	99(81.14%)	905(57.80%)	
T3a-b	415(24.58%)	15(12.30%)	400(25.54%)	
T4	269(15.94%)	8(6.56%)	261(16.66%)	
Clinical N Stage (n, %)				< 0.0001
N0	1224(72.51%)	109(89.34%)	1115(71.20%)	
N1	464(27.49%)	13(10.66%)	451(28.80%)	
Clinical M Stage (n, %)				0.1419
M0	1292(76.54%)	100(81.97%)	1192(76.12%)	
M1a-c	396(23.46%)	22(18.03%)	374(23.88%)	
[^18^F]FDG SUV_max_ (Median, IQR)	4.60 (3.00–7.20)	3.60 (2.50–6.30)	4.70 (3.00–7.20)	0.0046
[^68^Ga]Ga-PSMA-11 SUV_max_ (Median, IQR)	16.30 (9.10–27.50)	3.40 (2.63–4.10)	17.40 (10.50–28.90)	< 0.0001
[^68^Ga]Ga-PSMA-11 Diuretic Delayed SUV_max_ (Median, IQR)	24.30 (13.30–40.43)	4.40 (3.30–5.60)	25.85 (15.43–42.78)	< 0.0001

Together, these findings define low-PSMA HSPC as an early-arising and clinically distinct tumor subset that, despite presenting with features of lower tumor burden, retains a substantial proportion of high-grade prostate cancer and therefore cannot be considered a low-risk entity.

### Establishment of an imaging-pathology concordant cohort for proteomic profiling

**Fig. 2:**
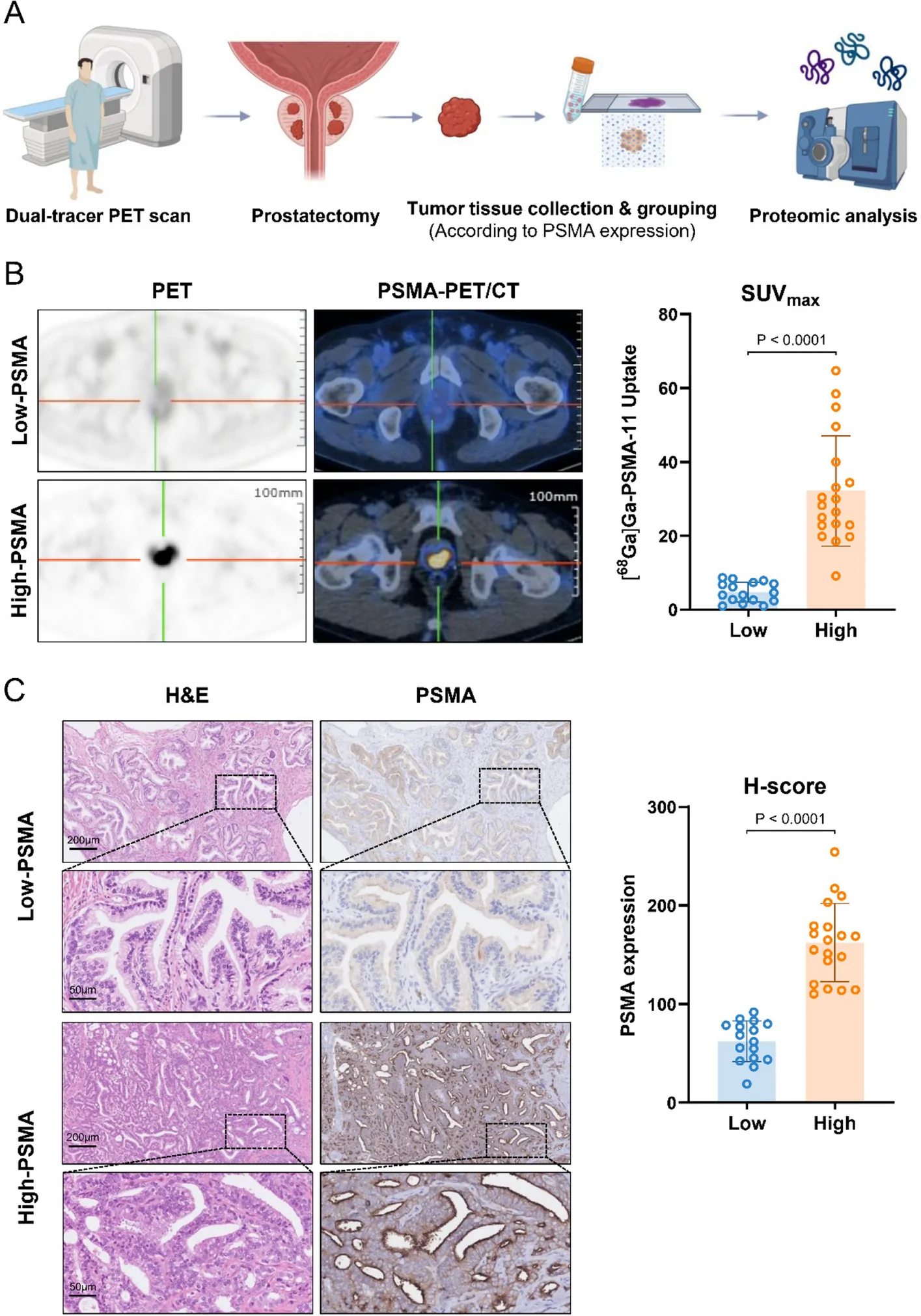
Establishment of the imaging–pathology matched proteomic cohort for low-PSMA and high-PSMA prostate cancer. **A** Schematic overview of the study workflow for the imaging–pathology matched proteomic cohort, including dual-tracer PET imaging, tumor tissue collection, pathological validation, and label-free quantitative proteomic profiling. **B** Representative [^68^Ga]Ga-PSMA-11 PET/CT images from low-PSMA and high-PSMA prostate cancer patients (left) and quantification of primary-tumor PSMA uptake (right), showing markedly reduced PSMA uptake in low-PSMA tumors. **C** Representative PSMA immunohistochemistry (IHC) staining of tumor tissues from low PSMA uptake and high PSMA uptake patients, with magnified insets (left), and quantitative comparison of PSMA H-scores between groups (right), confirming lower PSMA protein expression in low-PSMA tumors and concordance with PET findings.Statistical procedures are described in the Methods. *P* < 0.05 was considered statistically significant

To define the molecular basis of low-PSMA disease, we established a radical prostatectomy cohort for proteomic profiling. The overall study workflow, including preoperative dual-tracer PET imaging, surgical specimen collection, tissue processing, and downstream proteomic analysis, is summarized in Fig. 2A. A total of 35 patients with baseline dual-tracer PET and available surgical specimens were ultimately included, of whom 16 were classified as low-PSMA and 19 as high-PSMA (Supplementary Figure S1A; Table 2). In this cohort, the two groups were generally comparable in age, Gleason score distribution, and clinical T stage, whereas serum PSA remained significantly lower in the low-PSMA group.

**Table 2:** Baseline clinical characteristics of the proteomic cohort

Clinical Characteristics	Total	Low-PSMA	High-PSMA	*P* Value
	(*N* = 35)	(*N* = 16)	(*N* = 19)	
Age (y, Median, IQR)	67.00 (62.50–72.00)	66.00 (62.75–71.50)	68.00 (63.00–71.00)	0.7000
BMI (kg/m^2^, Median, IQR)	25.35 (23.93–27.12)	25.24 (23.18–27.02)	25.35 (23.91–27.05)	0.7499
Serum PSA (ng/ml, Median, IQR)	13.77 (8.68–32.50)	9.59 (7.42–16.21)	17.11 (11.50–35.18)	0.0450
Gleason Score (n, %)				0.1354
≤ 7	26(74.29%)	14(87.50%)	12(63.2%)	
≥ 8	9(25.71%)	2(12.50%)	7(36.8%)	
Clinical T Stage (n, %)				0.4605
T2a-c	26(74.29%)	13(81.25%)	13(68.4%)	
T3a-b	9(25.71%)	3(18.75%)	6(31.6%)	
T4	0(0%)	0(0%)	0(0%)	
[^18^F]FDG SUV_max_ (Median, IQR)	3.70 (3.00–6.65)	3.00 (2.15–3.63)	6.30 (3.80–8.25)	0.0012
[^68^Ga]Ga-PSMA-11 SUV_max_ (Median, IQR)	17.20 (4.30–29.1)	4.00 (2.20–6.85)	28.2 (22.60–33.73)	< 0.0001
[^68^Ga]Ga-PSMA-11 Diuretic Delayed SUV_max_ (Median, IQR)	23.70 (7.15–39.40)	6.35 (3.00–8.48)	39.4 (31.35–52.85)	< 0.0001

Imaging and tissue level analyses further supported the biological distinction between the two groups. Compared with high-PSMA tumors, low-PSMA tumors showed significantly lower [^68^Ga]Ga-PSMA-11 uptake on both routine and diuretic delayed imaging (Table 2; Fig. 2B). [^18^F]FDG uptake was also lower in the low-PSMA group (Table 2). Representative [^68^Ga]Ga-PSMA-11 PET images and corresponding PSMA immunohistochemical staining are shown in Fig. 2B-C. Consistent with the imaging findings, low-PSMA tumors exhibited markedly reduced tissue level PSMA expression, and PET-derived PSMA uptake was positively correlated with PSMA H-score across samples (Supplementary Figure S1B).

Together, these data demonstrate that low-PSMA status is a reproducible and biologically grounded phenotype across imaging and tissue levels, thereby providing a robust framework for subsequent proteomic interrogation.

### Proteomic profiling identifies a distinct tumor state associated with low PSMA expression

**Fig. 3:**
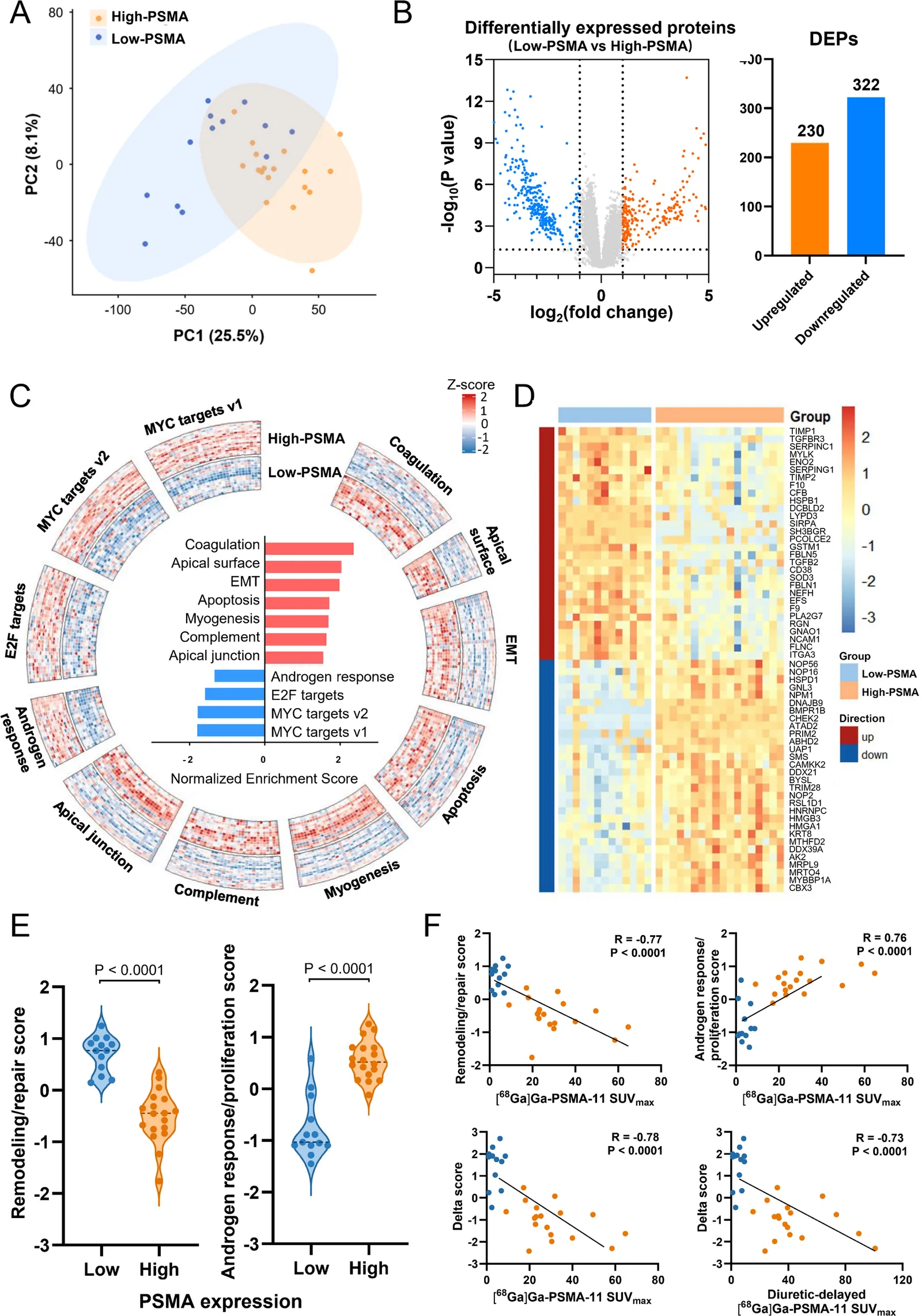
Proteomic characterization of low-PSMA prostate cancer reveals enhanced remodeling/repair features and attenuated androgen response/proliferation signaling. **A** Principal component analysis (PCA) of tumor proteomic profiles in the low-PSMA and high-PSMA groups, showing partial but discernible separation between the two groups and indicating global proteomic divergence. **B** Volcano plot of differentially expressed proteins (DEPs) between low-PSMA and high-PSMA tumors (left) and summary of significantly upregulated and downregulated proteins (right), highlighting extensive proteomic remodeling in low PSMA tumors. **C** Gene set enrichment analysis (GSEA) of proteomic data, with normalized enrichment scores (NES) for representative pathways shown in the central bar plot and pathway-level protein expression patterns displayed in the surrounding circular heatmaps, indicating distinct pathway activity states between low-PSMA and high-PSMA tumors. **D** Heatmap of representative core proteins derived from selected pathways, organized by group and direction of differential expression, illustrating coherent proteomic features that distinguish low-PSMA from high-PSMA tumors. **E** Violin plots comparing remodeling/repair and androgen response/proliferation scores between low-PSMA and high-PSMA tumors, showing higher remodeling/repair scores and lower androgen response/proliferation scores in low-PSMA tumors. Boxplots within violins indicate the median and interquartile range. **F** Correlation analyses between PSMA imaging parameters and pathway-derived scores, including remodeling/repair, androgen response/proliferation, and Delta (remodeling/repair minus androgen response/proliferation) scores, demonstrating associations between imaging phenotype and proteomic features. Statistical procedures are described in the Methods. *P* < 0.05 was considered statistically significant

We next asked whether low-PSMA tumors represent a distinct molecular state at the proteomic level. Label-free quantitative proteomic profiling was performed in imaging-pathology concordant tumors, including 16 low-PSMA expression samples (miPSMA 0–1 with low PSMA IHC) and 19 high-PSMA expression samples (miPSMA 2–3 with high PSMA IHC). Unsupervised principal component analysis showed a partial but discernible separation between the two groups, with low-PSMA tumors also displaying a trend toward greater intragroup dispersion in principal component analysis (PCA) space (Fig. 3A; Supplementary Figure S2A). Differential expression analysis further demonstrated extensive proteomic remodeling in low-PSMA tumors, including 230 upregulated and 322 downregulated proteins relative to high-PSMA tumors (Fig. 3B).

To define coordinated biological programs underlying these differences, we performed Hallmark pathway enrichment analysis across the full proteome. Low-PSMA tumors were positively enriched for coagulation (NES = 2.36, *P* < 0.001), apical surface (NES = 2.04, *P* < 0.001), EMT (NES = 1.99, *P* < 0.001), and apical junction (NES = 1.56, *P* = 0.003), whereas androgen response (NES = −1.34, *P* = 0.08), E2F targets (NES = −1.59, *P* = 0.04), and MYC targets (NES = −1.80, *P* < 0.001) were negatively enriched (Fig. 3C). Leading-edge proteins from these Hallmark signatures substantially overlapped with differentially expressed proteins (DEPs), supporting that the pathway-level shifts reflected coordinated protein-level alterations rather than isolated changes (Supplementary Figure S2B).

Based on these intersecting features, we derived a core protein panel that robustly distinguished low-PSMA from high-PSMA tumors at the sample level (Fig. 3D). We then summarized these proteomic features into two signature scores: a remodeling and repair-like score derived from features enriched in low-PSMA tumors, and an androgen response and proliferation-related score derived from features reduced in low-PSMA tumors. Consistent with this pattern, low-PSMA tumors showed significantly higher remodeling and repair-like scores and significantly lower androgen response and proliferation-related scores than high-PSMA tumors, while the composite Delta score further enhanced group separation (Fig. 3E; Supplementary Figure S2C). This separation was also evident in the two-dimensional signature space defined by these scores, in which low-PSMA and high-PSMA tumors occupied distinct regions (Supplementary Figure S2D). To assess the clinical relevance of these proteomic features, we next examined their associations with imaging and clinicopathological variables. Notably, PSMA SUV_max_ was negatively correlated with the remodeling and repair-like score and positively correlated with the androgen response and proliferation-related score, whereas the Delta score was negatively correlated with both standard and diuretic delayed PSMA SUV_max_ (Fig. 3F). Additional analyses showed that these signature scores were more strongly associated with PSMA PET uptake than with Gleason score or [^18^F]FDG SUV_max_ (Supplementary Figure S2E).

To assess whether these differences were primarily attributable to tissue composition, we compared stromal score, immune score, ESTIMATE score, and inferred tumor purity between groups. None of these metrics differed significantly between low PSMA and high PSMA expression samples, suggesting that the observed proteomic differences were unlikely to be driven mainly by broad variation in stromal or immune content or by inferred tumor purity (Supplementary Figure S2F-G).

Together, these data define low-PSMA prostate cancer as a distinct proteomic tumor state characterized by coordinated upregulation of structural and remodeling programs and attenuation of androgen response and proliferation-related signaling, with close coupling to PSMA imaging phenotypes.

### ALCAM is prioritized as a complementary surface marker associated with the low-PSMA tumor state

**Fig. 4:**
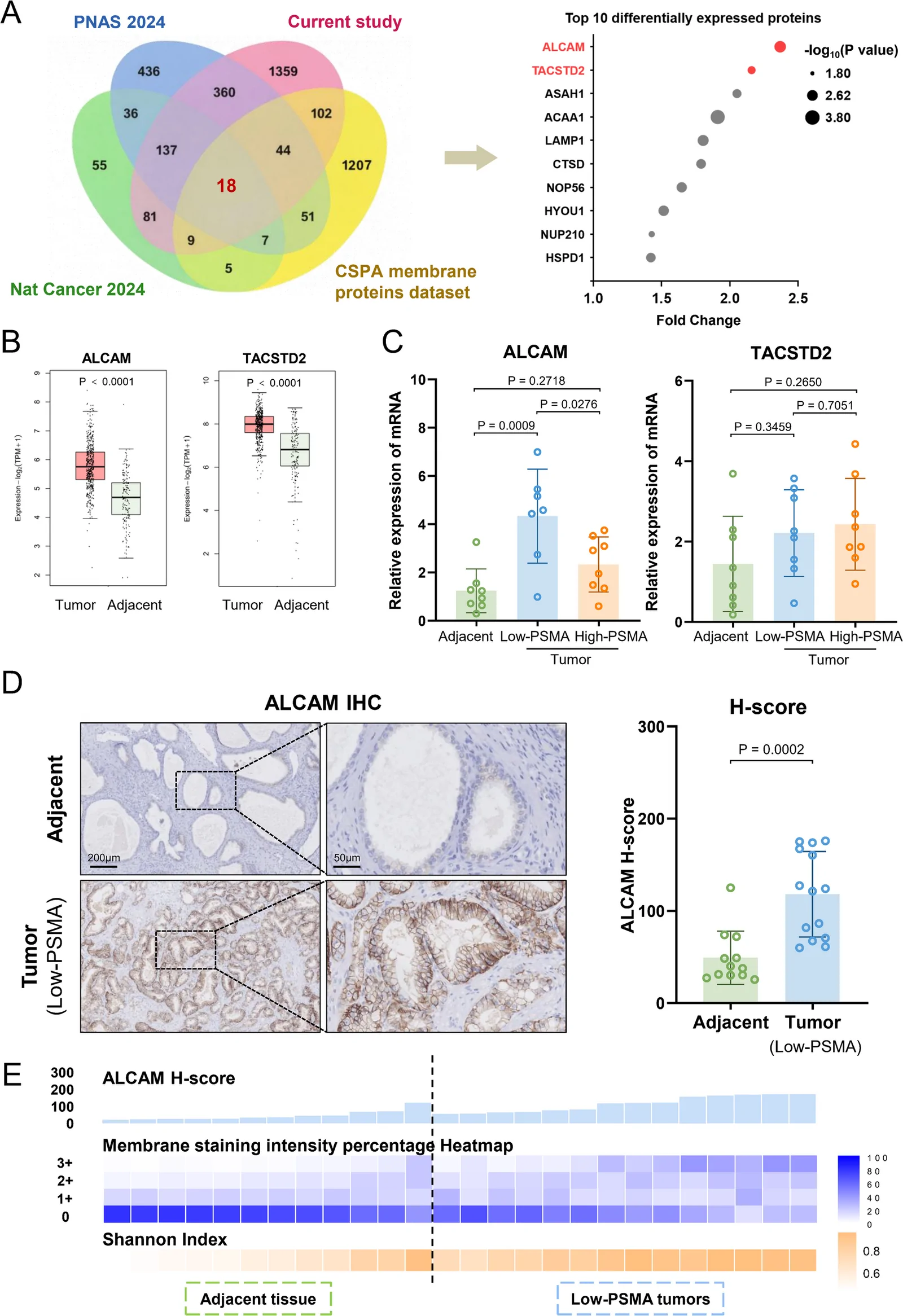
Identification and validation of ALCAM as a complementary membrane-associated target in low-PSMA prostate cancer. **A** Schematic illustration of the screening strategy for candidate membrane proteins. Proteins were filtered based on membrane localization and higher expression in low-PSMA tumors relative to adjacent normal tissues, resulting in the identification of 18 candidate proteins. **B** Transcriptomic validation in the TCGA-PRAD cohort showing that both ALCAM and TACSTD2 were significantly upregulated in tumor tissues compared with adjacent normal tissues. **C** Reverse transcription quantitative PCR (RT-qPCR) analysis of patient-derived samples showing increased ALCAM mRNA expression in low-PSMA hormone-sensitive prostate cancer (HSPC). **D** Representative IHC staining and quantitative analysis confirming increased ALCAM protein expression in tumor tissues from patients with low-PSMA HSPC. **E** Sample-wise distribution of ALCAM H-scores across adjacent and low-PSMA tissues, shown together with a membrane staining intensity heatmap and corresponding Shannon index. Samples are ordered by ALCAM H-score, and the dashed line indicates the boundary between adjacent and low-PSMA groups, highlighting increased membranous ALCAM expression and greater staining heterogeneity in low-PSMA tumors. Statistical procedures are described in the Methods. *P* < 0.05 was considered statistically significant

We next sought to identify membrane proteins that could complement PSMA-based detection in low-PSMA prostate cancer. Through an integrative screening strategy, DEPs from our proteomic comparison of low-PSMA versus adjacent benign tissues proteomic comparison were intersected with candidate targets reported in two independent studies (Dong et al. 2024; Wang et al. 2024) and with the cell surface protein atlas (CSPA) (Bausch-Fluck et al. 2015), yielding 18 overlapping membrane-associated proteins (Fig. 4A). Based on prior evidence, membrane localization, and expression consistency, ALCAM and TACSTD2 (TROP2) were prioritized for further evaluation.

Public transcriptomic data provided initial support for both candidates. In the TCGA-PRAD cohort, ALCAM and TACSTD2 were both significantly upregulated in tumor tissues relative to normal prostate tissues (Fig. 4B), while a broader overview of the remaining overlapping candidates is shown in Supplementary Figure S3A. In addition, pan-cancer transcriptomic profiling indicated that ALCAM is relatively abundant in prostate cancer compared with many other tumor types, further supporting its prioritization as a candidate surface marker in low-PSMA prostate cancer (Supplementary Figure S3B).

We then examined these candidates in our own tissue cohort comprising adjacent benign tissues, low-PSMA tumors, and high-PSMA tumors. At the transcript level, ALCAM expression was significantly elevated in low-PSMA tumors relative to adjacent tissues and remained higher than in high-PSMA tumors, whereas TACSTD2 expression did not differ significantly among groups (Fig. 4C). Consistent with the PSMA-based grouping, PSMA mRNA expression was markedly higher in high-PSMA tumors and substantially reduced in low-PSMA tumors (Supplementary Figure S3C).

Protein-level validation further strengthened the evidence supporting ALCAM. IHC analysis showed markedly stronger membranous ALCAM staining in low-PSMA tumors than in adjacent tissues, and H-score quantification confirmed significantly increased ALCAM protein expression in the low-PSMA group (Fig. 4D; Supplementary Figure S3D). This pattern was further supported by the distribution of ALCAM H-scores, a membrane staining intensity heatmap, and the Shannon index, all of which consistently indicated enhanced ALCAM-associated membrane staining in low-PSMA tumors (Fig. 4E).

Together, these findings identify ALCAM as a reproducibly upregulated membrane protein in low-PSMA prostate cancer, whereas TACSTD2 lacked consistent validation in our cohort. These findings nominate ALCAM as a candidate complementary surface biomarker for molecular characterization and alternative imaging strategies of low-PSMA prostate cancer.

## Discussion

Our study demonstrates that low-PSMA prostate cancer is not restricted to heavily treated or lineage-switched, but is already present in a definable subset of hormone-sensitive prostate cancer (HSPC) at initial diagnosis. This finding expands the current view of PSMA heterogeneity, which has largely been discussed in the context of metastatic castration resistant prostate cancer (mCRPC) (Paschalis et al. 2019; Pan et al. 2023; Robertson et al. 2020) and neuroendocrine prostate cancer (NEPC) (Puca et al. 2019; Bakht et al. 2018; Imberti et al. 2024). Instead our data support the concept that reduced PSMA expression represents an intrinsic dimension of prostate cancer biology rather than merely a late consequence of therapy pressure.

A major contribution of this study is the linkage of the low-PSMA imaging phenotype to a coherent proteomic state. Rather than simply reflecting reduced expression of a single surface target (Rüschoff et al. 2021), low-PSMA tumors exhibited coordinated enrichment of pathways associated with extracellular remodeling and structural organization, accompanied by attenuation of androgen-response, E2F-target, and MYC-target proliferative programs. These findings extend previous transcriptomic observations into an imaging-pathology-concordant proteomic framework and support the view that low-PSMA HSPC represents a biologically distinct tumor state rather than merely a low-uptake imaging variant (Weiner et al. 2024; Bakht et al. 2023). The enrichment of remodeling-related pathways may reflect altered epithelial organization, cell–cell adhesion, stromal interaction, and microenvironmental repair responses (Bakht et al. 2023; Romero et al. 2024; Han et al. 2022), whereas reduced androgen response and proliferation-associated signatures suggest diminished reliance on canonical AR-driven programs. Given the close relationship between prostate lineage signaling and PSMA expression (Al Jalali et al. 2023), attenuation of AR-related transcriptional activity may contribute to the low-PSMA phenotype and potentially influence sensitivity to AR-directed therapies.

Our findings also have direct implications for PSMA-based molecular characterization. Because a non-negligible subset of HSPC already exhibits reduced PSMA expression at diagnosis, complementary surface biomarkers may help refine the identification and biological interpretation of low-PSMA prostate cancer (Jochumsen and Bouchelouche 2024; Ozen et al. 2026; Bakht and Beltran 2024). Using a proteomics-based prioritization strategy, we identified ALCAM as a reproducibly upregulated membrane protein associated with the low-PSMA tumor state and validated its expression at both the mRNA and protein levels (Hansen et al. 2014; Sperger et al. 2023; Mulati et al. 2025). Although its functional significance and imaging utility require further investigation, ALCAM may serve as a candidate complementary surface biomarker for the molecular characterization of low-PSMA HSPC.

Low PSMA expression may have implications not only for disease detection but also for treatment-response assessment. In our recent dual-tracer PET study of advanced HSPC, PSMA PET provided distinct information at the patient and lesion levels: lower whole-body PSMA-derived tumor volume was associated with longer progression-free survival, whereas individual lesions with lower baseline [^68^Ga]Ga-PSMA-11 uptake showed less favorable short-term radiographic response to ADT-based systemic therapy (Li et al. 2026). This finding suggests that low-PSMA lesions may represent biologically distinct sites with reduced treatment sensitivity. Further prospective studies are needed to determine whether low-PSMA HSPC is associated with differential treatment response and long-term clinical outcomes.

The relationship between PSMA and[^18^F]FDG uptake in low-PSMA HSPC appears context-dependent rather than simply reciprocal. In our cohort, low-PSMA tumors exhibited lower rather than higher [^18^F]FDG uptake, suggesting that reduced PSMA expression in treatment-naive HSPC does not uniformly indicate a glycolytic phenotype. Consistently, our previous dual-tracer PET study showed that [^18^F]FDG uptake did not further stratify short-term radiographic response within either low-PSMA or high-PSMA lesions (Li et al. 2026). Nevertheless, discordant PSMA-negative/FDG-positive disease is associated with adverse outcomes, supporting the potential complementary value of [^18^F]FDG PET in selected patients (Chen et al. 2025). Future studies should clarify the prognostic, and treatment-response value of combined PSMA and[^18^F]FDG PET in low-PSMA HSPC.

Several limitations should be acknowledged. First, this was a retrospective single-center study, and because [^68^Ga]Ga-PSMA-11 PET was mainly performed in patients with high-risk disease at our institution, the observed frequency of low-PSMA HSPC may not represent its true prevalence in the overall newly diagnosed population. Second, the proteomic cohort was modest in size and based on radical prostatectomy specimens, reflecting a surgically selected population. Third, our molecular conclusions are based on proteomic associations rather than functional experiments. Finally, although ALCAM was reproducibly upregulated in low-PSMA tumors, its imaging or therapeutic utility requires further validation.

## Conclusion

In conclusion, low-PSMA hormone-sensitive prostate cancer (HSPC) represents a biologically distinct tumor state characterized by coordinated remodeling programs and attenuated androgen-response/proliferation signatures. This state is detectable at initial diagnosis and highlights the need for complementary molecular strategies to improve the characterization of tumors with reduced PSMA expression.

## Supplementary Information

Supplementary Material 1.

## Data Availability

The mass spectrometry proteomics dataset generated and analyzed during the current study has been deposited in the ProteomeXchange Consortium under the accession number PXD076661. Two external public proteomic datasets used and analyzed in this study are publicly available from PXD049446 and OEP004998.

## Abbreviations

PSMA: Prostate-specific membrane antigen
HSPC: Hormone-sensitive prostate cancer
CRPC: Castration resistant prostate cancer
NEPC: Neuroendocrine prostate cancer
PSA: Prostate-specific antigen
SUV_max_: Maximum standard uptake value
IHC: Immunohistochemistry
H&E: Hematoxylin and eosin
LC–MS/MS: Liquid chromatography-tandem mass spectrometry
FDR: False discovery rate
PCA: Principal component analysis
DEPs: Differentially expressed proteins
GSEA: Gene set enrichment analysis
GO: Gene ontology
KEGG: Kyoto encyclopedia of genes and genomes
TCGA-PRAD: The cancer genome atlas prostate adenocarcinoma
ORs: Odds ratios
Cis: Confidence intervals

## References

[CR1] Al Jalali V, Wasinger G, Rasul S, Grubmüller B, Wulkersdorfer B, Balber T, et al. Consecutive Prostate-Specific Membrane Antigen (PSMA) and Antigen Receptor (AR) PET Imaging Shows Positive Correlation with AR and PSMA Protein Expression in Primary Hormone-Naïve Prostate Cancer. J Nucl Med. 2023;64(6):863–8. 10.2967/jnumed.122.264981. 36657982

[CR2] Bakht MK, Beltran H. Aiming high: prostate-specific membrane antigen expression in treatment-naïve prostate cancer. Eur Urol. 2024;86(6):588–90. 10.1016/j.eururo.2024.09.033. 39394012 PMC11830280

[CR3] Bakht MK, Derecichei I, Li Y, Ferraiuolo RM, Dunning M, Oh SW, et al. Neuroendocrine differentiation of prostate cancer leads to PSMA suppression. Endocr Relat Cancer. 2018;26(2):131–46. 10.1530/ERC-18-0226. PubMed PMID: 30400059. 30400059

[CR4] Bakht MK, Yamada Y, Ku SY, Venkadakrishnan VB, Korsen JA, Kalidindi TM, et al. Landscape of prostate-specific membrane antigen heterogeneity and regulation in AR-positive and AR-negative metastatic prostate cancer. Nat Cancer. 2023;4(5):699–715. 10.1038/s43018-023-00539-6. 37038004 PMC10867901

[CR5] Bausch-Fluck D, Hofmann A, Bock T, Frei AP, Cerciello F, Jacobs A, et al. A Mass Spectrometric-Derived Cell Surface Protein Atlas. PLoS ONE. 2015;10(4):e0121314. 10.1371/journal.pone.0121314. 25894527 PMC4404347

[CR6] Bray F, Laversanne M, Sung H, Ferlay J, Siegel RL, Soerjomataram I, et al. Global cancer statistics 2022: GLOBOCAN estimates of incidence and mortality worldwide for 36 cancers in 185 countries. CA Cancer J Clin. 2024;74(3):229–63. 10.3322/caac.21834. PubMed PMID: 38572751. 38572751

[CR7] Chen R, Wang Y, Zhu Y, Shi Y, Xu L, Huang G, et al. The Added Value of 18F-FDG PET/CT Compared with 68Ga-PSMA PET/CT in Patients with Castration-Resistant Prostate Cancer. J Nucl Med. 2022;63(1):69–75. 10.2967/jnumed.120.262250. PubMedPMID:34980667; PubMedCentralPMCID: PMC8717199. 34980667 PMC8717199

[CR8] Chen R, Wang Y, Chen Q, Dong L, Huang G, Liu J. Dual-tracer PET/CT identifies an aggressive PSMA-negative/FDG-positive phenotype predicting early failure of radical prostatectomy in treatment-naive prostate cancer. Eur J Nucl Med Mol Imaging. 2025. 10.1007/s00259-025-07646-9. 41212260

[CR9] Chen R, Wang Y, Dong L, Zhao H, Li L, Huang G, et al. A dual-tracer approach for enhanced nodal staging in prostate cancer: the role of [18F]FDG PET/CT for detecting [68 Ga]Ga-PSMA-11-negative metastases. Eur J Nucl Med Mol Imaging. 2025. 10.1007/s00259-025-07683-4 41296030

[CR10] Dardenne E, Beltran H, Benelli M, Gayvert K, Berger A, Puca L, et al. N-Myc Induces an EZH2-Mediated Transcriptional Program Driving Neuroendocrine Prostate Cancer. Cancer Cell. 2016;30(4):563–77. 10.1016/j.ccell.2016.09.005. PubMedPMID: 27728805; PubMedCentralPMCID: PMC5540451. 27728805 PMC5540451

[CR11] Derlin T, Riethdorf S, Schumacher U, Lafos M, Peine S, Coith C, et al. PSMA-heterogeneity in metastatic castration-resistant prostate cancer: Circulating tumor cells, metastatic tumor burden, and response to targeted radioligand therapy. Prostate. 2023;83(11):1076–88. 10.1002/pros.24549. PubMed PMID: 37147881. 37147881

[CR12] Dong B, Xu JY, Huang Y, Guo J, Dong Q, Wang Y, et al. Integrative proteogenomic profiling of high-risk prostate cancer samples from Chinese patients indicates metabolic vulnerabilities and diagnostic biomarkers. Nat Cancer. 2024;5(9):1427–47. 10.1038/s43018-024-00820-2. 39242942

[CR13] Ergül N, Çermik TF, Alçın G, Arslan E, Erol Fenercioğlu Ö, Beyhan E, et al. Contribution of 68Ga-DOTA-FAPI-04 PET/CT to prostate cancer imaging: complementary role in PSMA-negative cases. Clin Nucl Med. 2024;49(3):e105–10. 10.1097/RLU.0000000000005064. 38271254

[CR15] Ferraro DA, Rüschoff JH, Muehlematter UJ, Kranzbühler B, Müller J, Messerli M, et al. Immunohistochemical PSMA expression patterns of primary prostate cancer tissue are associated with the detection rate of biochemical recurrence with ^68^Ga-PSMA-11-PET. Theranostics. 2020;10(14):6082–94. 10.7150/thno.44584 PMC7255040 32483440

[CR16] Gan Q, Cui K, Cao Q, Zhang N, Yang MF, Yang X. Development of a 18F-Labeled Bicyclic Peptide Targeting EphA2 for Molecular Imaging of PSMA-Negative Prostate Cancer. J Med Chem. 2023;66(21):14623–32. 10.1021/acs.jmedchem.3c01135. 37908059

[CR17] Han H, Wang Y, Curto J, Gurrapu S, Laudato S, Rumandla A, et al. Mesenchymal and stem-like prostate cancer linked to therapy-induced lineage plasticity and metastasis. Cell Reports. 2022;39(1). 10.1016/j.celrep.2022.110595 PubMed PMID: 35385726. PMC9414743 35385726

[CR18] Hansen AG, Arnold SA, Jiang M, Palmer TD, Ketova T, Merkel A, et al. ALCAM/CD166 is a TGF-β-responsive marker and functional regulator of prostate cancer metastasis to bone. Cancer Res. 2014;74(5):1404–15. 10.1158/0008-5472.CAN-13-1296. PubMedPMID:24385212; PubMedCentralPMCID: PMC4149913. 24385212 PMC4149913

[CR19] Humphrey PA. Gleason grading and prognostic factors in carcinoma of the prostate. Mod Pathol. 2004;17(3):292–306. 10.1038/modpathol.3800054. PubMed PMID: 14976540. 14976540

[CR20] Imberti C, De Gregorio R, Korsen JA, Hoang TT, Khitrov S, Kalidindi T, et al. CEACAM5-Targeted Immuno-PET in Androgen Receptor-Negative Prostate Cancer. J Nucl Med. 2024;65(7):1043–50. 10.2967/jnumed.123.267107. 38782457 PMC11218725

[CR21] Incesu RB, Preisser F, Nohe F, Maurer T, Graefen M, Tilki D. Negative PSMA PET can be used to avoid unnecessary pelvic lymph node dissection in intermediate risk prostate cancer. Prostate Cancer Prostatic Dis. 2025;28(4):874–8. 10.1038/s41391-024-00930-z. PubMed PMID: 39762549; PubMed Central PMCID: PMC12643919. 39762549 PMC12643919

[CR22] Jochumsen MR, Bouchelouche K. PSMA PET/CT for primary staging of prostate cancer - an updated overview. Semin Nucl Med. 2024;54(1):39–45. 10.1053/j.semnuclmed.2023.07.001. Prostate cancer. 37487824

[CR23] Kiess AP, Banerjee SR, Mease RC, Rowe SP, Rao A, Foss CA, et al. Prostate-specific membrane antigen as a target for cancer imaging and therapy. Q J Nucl Med Mol Imaging. 2015;59(3):241–68. PubMed PMID: 26213140; PubMed Central PMCID: PMC4859214. PMC4859214 26213140

[CR24] Li A, Wu H, Zhou X, Zhu Y, Zhang W, Shen K, et al. Distinct prognostic value of [18F]FDG PET and [68Ga]Ga-PSMA-11 PET in advanced hormone-sensitive prostate cancer. Commun Med (Lond). 2026;6(1):164. 10.1038/s43856-026-01444-6. PubMedPMID:41703276; PubMedCentralPMCID: PMC13022180. 41703276 PMC13022180

[CR25] Mulati Y, Shen Q, Tian Y, Chen Y, Yao K, Yu W, et al. Characterizing PSMA heterogeneity in prostate cancer and identifying clinically actionable tumor associated antigens in PSMA negative cases. Sci Rep. 2025;4(15):23902. 10.1038/s41598-025-06393-z. PubMed PMID: 40615466. PMC12227638 40615466

[CR26] Ozen A, Sarikaya Z, Gur C, Ozboru D, Canat HL. Pelvic Lymph Node Staging With PSMA PET in Prostate Cancer: Surgical Validation With Implications for Radiotherapy Planning. Prostate. 2026;86(6):648–54. 10.1002/pros.70124. PubMed PMID: 41570172. 41570172

[CR27] Pan J, Zhao J, Ni X, Zhu B, Hu X, Wang Q, et al. Heterogeneity of [68Ga]Ga-PSMA-11 PET/CT in metastatic castration-resistant prostate cancer: genomic characteristics and association with abiraterone response. Eur J Nucl Med Mol Imaging. 2023;50(6):1822–32. 10.1007/s00259-023-06123-5. PubMed PMID: 36719427. 36719427

[CR28] Parker CC, James ND, Brawley CD, Clarke NW, Hoyle AP, Ali A, et al. Radiotherapy to the primary tumour for newly diagnosed, metastatic prostate cancer (STAMPEDE): a randomised controlled phase 3 trial. Lancet. 2018;392(10162):2353–66. 10.1016/S0140-6736(18)32486-3. PubMedPMID:30355464; PubMedCentralPMCID:PMC6269599. 30355464 PMC6269599

[CR29] Paschalis A, Sheehan B, Riisnaes R, Rodrigues DN, Gurel B, Bertan C, et al. Prostate-specific Membrane Antigen Heterogeneity and DNA Repair Defects in Prostate Cancer. Eur Urol. 2019;76(4):469–78. 10.1016/j.eururo.2019.06.030. PubMedPMID:31345636; PubMedCentralPMCID: PMC6853166. 31345636 PMC6853166

[CR30] Puca L, Gavyert K, Sailer V, Conteduca V, Dardenne E, Sigouros M, et al. Delta-like protein 3 expression and therapeutic targeting in neuroendocrine prostate cancer. Sci Transl Med. 2019 Mar 20;11(484):eaav0891. 10.1126/scitranslmed.aav0891 PubMed PMID: 30894499; PubMed Central PMCID: PMC6525633. PMC6525633 30894499

[CR31] Qin J, Liu X, Laffin B, Chen X, Choy G, Jeter CR, et al. The PSA−/lo Prostate Cancer Cell Population Harbors Self-Renewing Long-Term Tumor-Propagating Cells that Resist Castration. Cell Stem Cell. 2012;10(5):556–69. 10.1016/j.stem.2012.03.009. PubMed PMID: 22560078. 22560078 PMC3348510

[CR32] Robertson MS, Sakellis CG, Hyun H, Jacene HA. Extraprostatic uptake of^18^ F-fluciclovine: differentiation of nonprostatic neoplasms from metastatic prostate cancer. Am J Roentgenol. 2020;214(3):641–8. 10.2214/AJR.19.21894. 31939697

[CR33] Romero R, Chu T, González Robles TJ, Smith P, Xie Y, Kaur H, et al. The neuroendocrine transition in prostate cancer is dynamic and dependent on ASCL1. Nat Cancer. 2024;5(11):1641–59. 10.1038/s43018-024-00838-6. 39394434 PMC11584404

[CR34] Rüschoff JH, Ferraro DA, Muehlematter UJ, Laudicella R, Hermanns T, Rodewald AK, et al. What’s behind 68Ga-PSMA-11 uptake in primary prostate cancer PET? Investigation of histopathological parameters and immunohistochemical PSMA expression patterns. Eur J Nucl Med Mol Imaging. 2021;48(12):4042–53. 10.1007/s00259-021-05501-1. 34386839 PMC8484204

[CR35] Sartor O, de Bono J, Chi KN, Fizazi K, Herrmann K, Rahbar K, et al. Lutetium-177-PSMA-617 for Metastatic Castration-Resistant Prostate Cancer. N Engl J Med. 2021;385(12):1091–103. 10.1056/NEJMoa2107322. PubMedPMID: 34161051;PubMed CentralPMCID:PMC8446332. 34161051 PMC8446332

[CR36] Sayar E, Patel RA, Coleman IM, Roudier MP, Zhang A, Mustafi P, et al. Reversible epigenetic alterations mediate PSMA expression heterogeneity in advanced metastatic prostate cancer. JCI Insight. 2023;8(7):e162907. 10.1172/jci.insight.162907. PubMedPMID:36821396; PubMedCentralPMCID:PMC10132157. 36821396 PMC10132157

[CR37] Shen K, Liu B, Zhou X, Ji Y, Chen L, Wang Q, et al. The Evolving Role of 18F-FDG PET/CT in Diagnosis and Prognosis Prediction in Progressive Prostate Cancer. Front Oncol. 2021;11:683793. 10.3389/fonc.2021.683793. PubMedPMID:34395251;PubMedCentralPMCID: PMC8358601. 34395251 PMC8358601

[CR38] Sperger JM, Helzer KT, Stahlfeld CN, Jiang D, Singh A, Kaufmann KR, et al. Expression and Therapeutic Targeting of TROP-2 in Treatment-Resistant Prostate Cancer. Clin Cancer Res. 2023;29(12):2324–35. 10.1158/1078-0432.CCR-22-1305. PubMedPMID:36939530; PubMedCentralPMCID: PMC10261916. 36939530 PMC10261916

[CR39] Sweeney CJ, Chen YH, Carducci M, Liu G, Jarrard DF, Eisenberger M, et al. Chemohormonal Therapy in Metastatic Hormone-Sensitive Prostate Cancer. N Engl J Med. 2015;373(8):737–46. 10.1056/NEJMoa1503747. PubMedPMID:26244877; PubMedCentralPMCID:PMC4562797. 26244877 PMC4562797

[CR40] Wang Z, Yu H, Bao W, Qu M, Wang Y, Zhang L, et al. Proteomic and phosphoproteomic landscape of localized prostate cancer unveils distinct molecular subtypes and insights into precision therapeutics. Proc Natl Acad Sci U S A. 2024;121(40):e2402741121. 10.1073/pnas.2402741121. 39320917 PMC11459144

[CR41] Weiner AB, Agrawal R, Wang NK, Sonni I, Li EV, Arbet J, et al. Molecular Hallmarks of Prostate-specific Membrane Antigen in Treatment-naïve Prostate Cancer. Eur Urol. 2024;86(6):579–87. 10.1016/j.eururo.2024.09.005. 39294048 PMC11637967

[CR42] Woo S, Becker AS, Leithner D, Mayerhoefer ME, Friedman KP, Tong A, et al. Discordance between prostate MRI and PSMA-PET/CT: the next big challenge for primary prostate tumor assessment? Eur Radiol. 2025;35(7):4043–54. 10.1007/s00330-025-11358-x. PubMed PMID: 39853335. 39853335

