# Supplementary material for: Low-PSMA hormone-sensitive prostate cancer: a distinct clinical and molecular state
Source: Mol Med. 2026 Jul 3;32:157. doi: 10.1186/s10020-026-01546-w (PMC13599339; doi:10.1186/s10020-026-01546-w)
Supplement: Supplementary file 1 [file 10020_2026_1546_MOESM1_ESM.docx]

**Supplementary Figure 1. Selection of the proteomic cohort and concordance between PSMA PET uptake and tissue PSMA expression. (A)** Flow diagram of patient inclusion and exclusion for the proteomic cohort. **(B)** Correlation analysis between PSMA H-score and PSMA PET uptake (SUVmax) in the final cohort, showing a positive association between tissue PSMA protein expression and imaging-based PSMA signal. Statistical procedures are described in the Methods. P < 0.05 was considered statistically significant.

**Supplementary Figure 2. Supplementary proteomic and tumor purity analyses of low-PSMA and high-PSMA prostate cancer**

**(A)** Comparison of the distance of each sample to the group centroid in PCA space (PC1–PC2) between low-PSMA and high-PSMA tumors, showing a trend toward greater intragroup dispersion in low-PSMA tumors.

**(B)** Bubble plot summarizing the overlap between leading-edge proteins and DEPs across representative Hallmark pathways, showing that both low-PSMA-up and low-PSMA-down features are supported by substantial concordant protein-level alterations. Bubble size indicates overlap count, and the x-axis indicates the proportion of leading-edge proteins overlapping with DEPs.

**(C)** Violin plots comparing the Delta score (Remodeling/Repair minus AR/Proliferation) between low-PSMA and high-PSMA tumors, showing markedly higher Delta scores in low-PSMA tumors. Boxplots within violins indicate the median and interquartile range.

**(D)** Two-dimensional distribution of tumors according to Remodeling/Repair and AR/Proliferation scores, showing clear separation of low-PSMA and high-PSMA tumors in protein-feature space.

**(E)** Bubble plot summarizing correlations between pathway-derived scores and clinicopathologic or imaging variables, showing the strongest associations between PSMA uptakes with these proteomic scores. Bubble color indicates the Spearman correlation coefficient, and bubble size indicates -log_10_(FDR).

**(F)** Violin plots comparing stromal score, immune score, and ESTIMATE score between low-PSMA and high-PSMA tumors, showing no significant differences in these purity-related metrics between groups. Boxplots within violins indicate the median and interquartile range.

**(G)** Tumor purity Comparison between low-PSMA and high-PSMA tumors, showing no significant difference between groups.

Statistical procedures are described in the Methods. P < 0.05 was considered statistically significant.

**Supplementary Figure 3. Additional transcriptomic and histologic validation of candidate proteins and PSMA expression.**

**(A)** Box plots showing the expression of representative selected genes in the TCGA PRAD cohort, comparing tumor and adjacent normal tissues, and indicating that multiple candidates are upregulated in prostate cancer tissues.

**(B)** Pan-cancer transcriptomic profiling of ALCAM across tumor types, with PRAD highlighted, showing its relative expression pattern in prostate cancer compared with other malignancies.

**(C)** RT-qPCR analysis of FOLH1 expression in adjacent tissues, low-PSMA tumors, and high-PSMA tumors, showing reduced FOLH1 expression in low-PSMA tumors relative to high-PSMA tumors.

**(D)** Representative hematoxylin and eosin (H&E) images of adjacent prostate tissue and low-PSMA tumor tissue, illustrating the corresponding histologic features of the analyzed samples.

Statistical procedures are described in the Methods. P < 0.05 was considered statistically significant.
